# Adsorption of Silver Nanoparticles onto Different Surface Structures of Chitin/Chitosan and Correlations with Antimicrobial Activities

**DOI:** 10.3390/ijms160613973

**Published:** 2015-06-18

**Authors:** Masayuki Ishihara, Vinh Quang Nguyen, Yasutaka Mori, Shingo Nakamura, Hidemi Hattori

**Affiliations:** 1 Research Institute, National Defense Medical College, Saitama 359-1324, Japan; E-Mails: snaka@ndmc.ac.jp (S.N.); h2@ndmc.ac.jp (H.H.); 2 Kobe VN Beef Corporation, Bao Loc City 670000, Vietnam; E-Mail: imvinhs@yahoo.com.vn; 3 Department of Applied Chemistry and Chemical Engineering National Institute of Technology, Toyama College, Toyama 939-8630, Japan; E-Mail: yamori@nc-toyama.ac.jp

**Keywords:** silver nanoparticles, chitin, chitosan, surface structure, antimicrobial activity

## Abstract

Size-controlled spherical silver nanoparticles (Ag NPs) can be simply prepared by autoclaving mixtures of glass powder containing silver with glucose. Moreover, chitins with varying degrees of deacetylation (DDAc < 30%) and chitosan powders and sheets (DDAc > 75%) with varying surface structure properties have been evaluated as Ag NP carriers. Chitin/chitosan-Ag NP composites in powder or sheet form were prepared by mixing Ag NP suspensions with each of the chitin/chitosan-based material at pH 7.3, leading to homogenous dispersion and stable adsorption of Ag NPs onto chitin carriers with nanoscale fiber-like surface structures, and chitosan carriers with nanoscale porous surface structures. Although these chitins exhibited mild antiviral, bactericidal, and antifungal activities, chitin powders with flat/smooth film-like surface structures had limited antimicrobial activities and Ag NP adsorption. The antimicrobial activities of chitin/chitosan-Ag NP composites increased with increasing amounts of adsorbed Ag NPs, suggesting that the surface structures of chitin/chitosan carriers strongly influence adsorption of Ag NPs and antimicrobial activities. These observations indicate that chitin/chitosan-Ag NPs with nanoscale surface structures have potential as antimicrobial biomaterials and anti-infectious wound dressings.

## 1. Introduction

Chitin/chitosan is the second most abundant polysaccharide in exoskeletons of seafood such as crab and shrimp, which are referred to as de-*N*-acetylated chitins with varying degrees of deacetylation (DDAc) [1,2]. Owing to their biological activity and safety, these compounds have attracted considerable interest as biomaterial sources for hydrogels, micro/nanoparticles, and membranes and sheets. In previous studies, chitin/chitosan was distinguished according to proportions of *N*-acetyglucosamine in the biopolymer with greater or less than 50% *N*-acetylglucosamine content, respectively. Chitin/chitosan has been widely studied as natural cationic biopolymer because of its excellent biocompatibility, biodegradability, nontoxicity [3], antimicrobial [4], tissue adhesive [5,6], hemostatis [7,8], and wound healing [9,10] properties.

Silver nanoparticles (Ag NPs) also have great potential as catalysts in various applications, including photonic devices, biosensors, antimicrobials, and drug delivery systems [11,12,13]. Accordingly, multiple processes have been reported for controlling the physical and/or chemical characteristics of Ag NPs [14,15,16,17], and environment-friendly processes have been devised using harmless materials to prepare Ag NPs, precluding the need for complicated purification procedures prior to use in biomedical and environmental applications [18,19]. We also reported the synthesis of <10 nm AG NPs using only AgNO_3_-containing glass powder, glucose, and water [20]. AgNO_3_-containing glass is usually used as an antimicrobial agent in environmental, osseous, or dental applications because it allows the sustained release of Ag^+^ into aqueous environments. Moreover, caramel formed by heating glucose functions as a stabilizing agent during preparation of these Ag NPs [20].

Biological and environmental risks of synthetic Ag NPs include adverse effects on some aquatic organisms, including cytotoxicity and genotoxicity in fish [21] and inhibition of photosynthesis in algae [22]. In addition, significant declines in mouse spermatogenic stem cells were observed following treatments with Ag NPs [23]. Therefore, methods for preventing the diffusion of Ag NPs into the environment and their uptake into human bodies are necessary before wide use as antimicrobial agents [21,22,23]. In recent studies, Ag NPs were efficiently adsorbed onto chitin powders and sheets with nanoscale fiber-like or chitosan powders with porous surface structures. In these applications, chitin/chitosan stabilized Ag NPs and caramel was no longer needed. The resulting chitin/chitosan-Ag NP composites showed stability and exhibited much stronger antimicrobial (antiviral, bactericidal, and antifungal) activities. Hence, introduction of small amounts of Ag NPs onto chitin/chitosan-based materials can substantially enhance the antimicrobial activities of Ag NPs [20,24,25,26]. In addition, it has been reported that a monolayer of Ag NPs anchored to an amino-sianized glass surface has strong antibiofilm activity [27].

Properties of chitin/chitosan are primarily dependent on molecular weight, DDAc, and their conformational structure [1,2]. DDAc especially affects solubility, hydrophobicity, and electrostatic interactions between polyanions and protonated amino groups of chitin/chitosan [1,2]. However, surface structures of chitin/chitosan carriers influence Ag NP adsorption, and play more important roles in the antimicrobial activities of chitin/chitosan-Ag NPs than the molecular structures of Ag NPs [28]. In the present review, we describe interactions of Ag NPs with chitin/chitosan powders with unique surface structures and chitin nanofiber sheets, and summarize previous evaluations of antimicrobial activities of chitin/chitosan-Ag NPs.

## 2. Preparation and Characterization of Chitin/Chitosan and Ag NPs Composites

Several preparation processes have been proposed for controlling the physical and/or chemical characteristics of Ag NPs [14,15,16,17], and particle sizes of Ag NPs are fundamental to their optical and antimicrobial properties [12,13]. Recently, environmentally innocuous processes that employ harmless materials have been utilized to prepare Ag NPs, and these have effectively circumvented the need for complicated purification procedures that are disadvantageous in industrial, biomedical, and environmental applications. Most of those processes involve reduction of Ag^+^ using non-toxic reducing and stabilizing agents in aqueous solutions. Moreover, Ag NPs with diameters of less than 10 nm have been produced using glucose as a reducing agent and soluble starch as a stabilizing agent [18]. Ag NP particle sizes are often controlled by modifying reaction system parameters such as pH, temperature, and reactant concentrations. However, stabilizing agents also influence particle sizes of Ag NPs, because Ag^+^ is reduced within their nanoscale templates [19]. Although various methods for synthesizing Ag NPs have been investigated, control of Ag NPs within narrow particle size distributions remains difficult [17,18,19].

**Figure 1:**
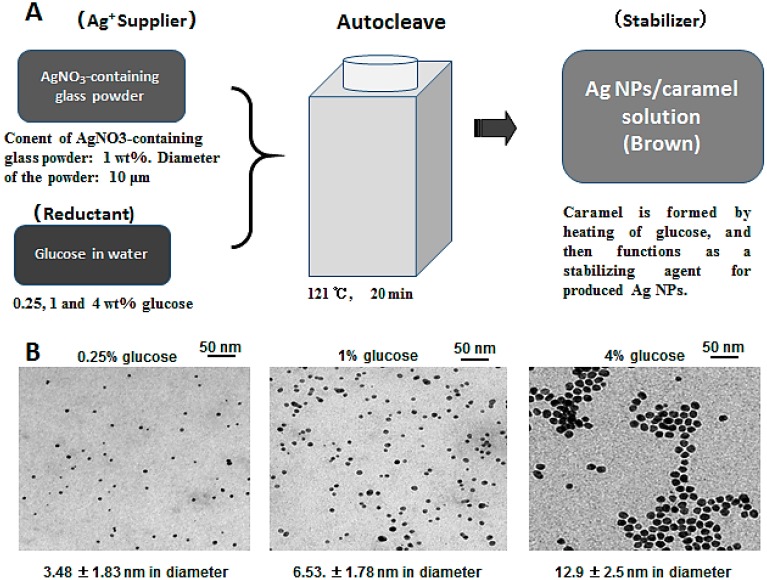
Synthesis of silver nanoparticles. (**A**) Environment-friendly processes were used to produce Ag NPs within a narrow size distribution; (**B**) Diameters of Ag NPs were controlled by glucose (reductant) concentrations. Currently, 0.8% glucose is used as a reductant, in which the generated Ag NPs are 5.17 ± 1.92 nm.

We previously demonstrated an environment-friendly process for producing Ag NPs within a narrow size distribution [20] using AgNO_3_-containing glass powder (BSP21, silver content, 1% wt; grain size, 10 μm; Kankyo Science, Kyoto, Japan), glucose, and water (Figure 1). AgNO_3_-containing glass powder is commonly used as an antimicrobial agent in environmental, osseous, and dental applications, and achieves sustained release of silver ions (Ag^+^) into aqueous environments. Glucose is environmentally innocuous and acts as a mild reducing agent, enabling easy control of reaction kinetics. In our studies, Ag NPs were synthesized in aqueous solutions using an autoclave at 121 °C and 200 kPa for 20 min (Figure 1). Caramel was formed during autoclaving, and contributed as a stabilizing agent for Ag NPs in this system [20]. Particle sizes were dependent on glucose concentrations, with 0.25, 1.0, and 4.0% wt glucose providing small (3.48 ± 1.83 nm in diameter), medium (6.53 ± 1.78 nm) and large (12.9 ± 2.5 nm) particles, respectively, each with yields of about 70% (Figure 1). However, it was difficult to remove the caramel from Ag NP suspensions without agglomeration and precipitation of Ag NPs. Thus, additions of chitin/chitosan were necessary to remove caramels and acted as alternative carriers and stabilizers for Ag NPs. Moreover, chitin/chitosan-Ag NPs composites were stable even after washing several times with water and drying [20,26,28].

**Figure 2:**
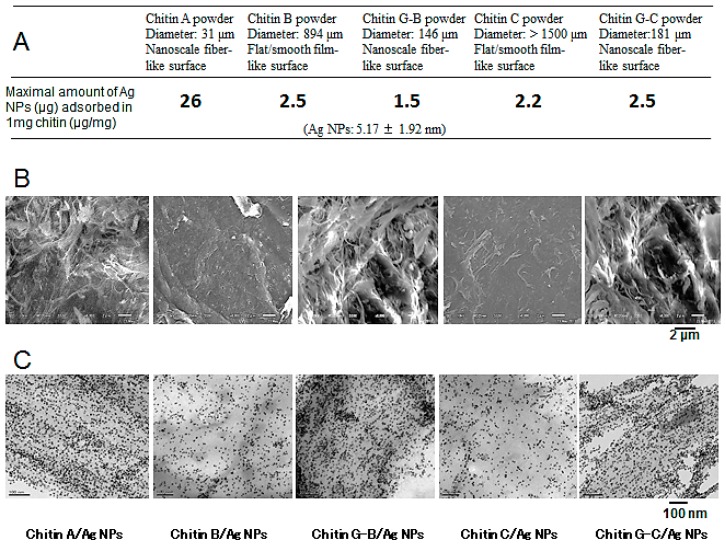
Interactions of Ag NPs with chitins comprising nanoscale fiber-like or flat/smooth film-like surface structures. (**A**) Binding of Ag NPs onto each chitin; (**B**) SEM micrographs of each chitin powder; (**C**) TEM micrographs of each chitin-Ag NP composite. A total of 1mg of each chitin was added to a 10 μg/mL suspension of Ag NPs and mixed for 30 min prior to analysis.

In a previous study [28], chitins A, B, ground B (G-B), C and ground C (G-C) with DDAc of <5%, various powder sizes, and different surface structures were added to stabilize Ag NP and to remove the caramel that was produced during autoclaving. Chitins G-B and G-C were prepared using a grinding machine under acidic conditions (0.15 M acetic acid). Ag NPs were tightly adsorbed to chitin A by simply mixing at pH 7.3 for 30 min (Figure 2A). As shown in Figure 2A, approximately 26, 2.5, 20, 1.5, and 18 μg of Ag NPs were maximally adsorbed to 1 mg of chitins A, B, G-B, C, and G-C, respectively. Moreover, caramel was removed from chitin-Ag NP composites by washing twice with distilled water. Removal of caramel was important because of its stimulatory effect on microbial growth [26,28,29].

Typical Scanning Electron Microscope (SEM) micrographs of chitins A, B, G-B, C, and G-C are shown in Figure 2B. Chitins A, G-B, and G-C exhibited smaller powder sizes and nanoscale fiber-like surface structures than chitins B and C, which showed larger powder sizes and flat/smooth film-like surface structures. In particular, chitins G-B and G-C exhibited smaller powder sizes and nanoscale fiber-like surface structures that were similar to those of chitin A (Figure 2B). The sizes and shapes of Ag NPs adsorbed to the present chitins were identical to those of the original Ag NPs. Transmission Electron Microscope (TEM) micrographs showed efficient binding of Ag NPs to chitins A, G-B, and G-C (Figure 2C), and the average powder sizes of chitins G-B and G-C were 146 ± 62 and 181 ± 52 μm, respectively (Figure 2A), indicating that larger chitin powders with flat/smooth film-like surface structures can be converted into smaller particles with nanoscale fiber-like surface structures by grinding under acidic conditions. The present composites were brown in color, and darker composites were produced with adsorption of larger amounts of Ag NPs [26,28,29].

**Figure 3:**
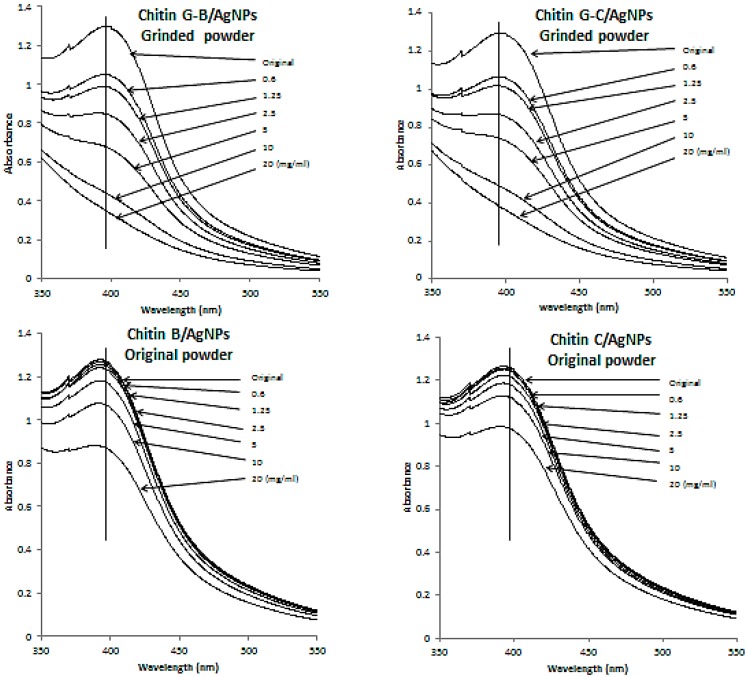
UV-Vis spectra of original Ag NPs in suspension (original) and supernatants of postreaction mixtures in which various amounts of chitins G-B, B, G-C, or C were reacted with Ag NPs. Excess Ag NPs in the supernatants of postreaction mixtures decreased as the amount of chitin added increased.

UV-Vis spectra from Ag NPs in suspension and supernatants of post-reaction mixtures showed variations in the amounts of chitin that reacted with Ag NPs. Moreover, a peak at 390.5 nm was representative of the spherical Ag NPs used in this work (Figure 3) [26], and the amounts of Ag NPs remaining in supernatants of post-reaction mixtures decreased with increasing concentrations of chitin. Thus, Ag NPs showed selective reactions with chitins G-B, G-C, and chitin A, and limited reactions with chitins B and C [28].

**Figure 4:**
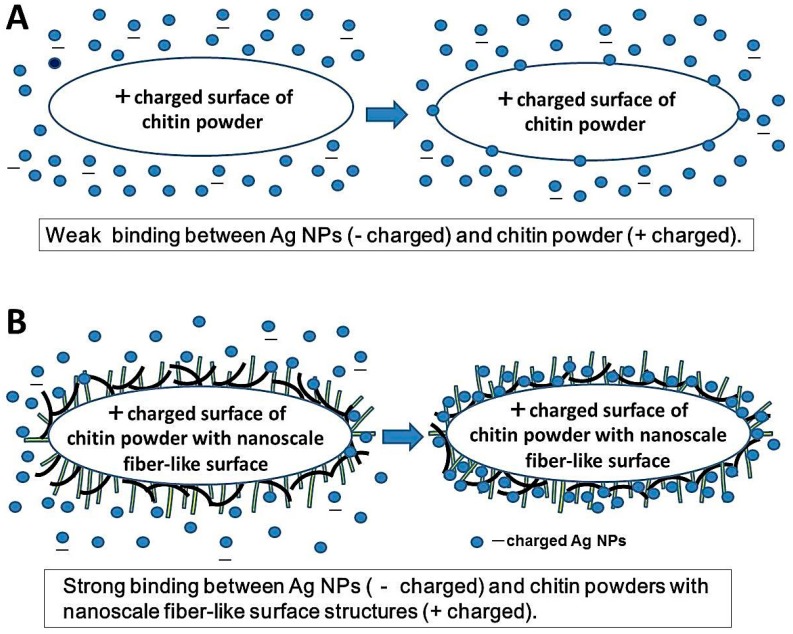
Scheme on adsorption of Ag NPs onto chitin with flat/smooth film-like surface structures (**A**) with nanoscale fiber-like surface structures (**B**).

The scheme in Figure 4 shows adsorption of Ag NPs onto chitin powders with flat/smooth film-like surface structures or nanoscale fiber-like surface structures. Ag NPs bind more strongly and stably to chitins with nanoscale fiber-like surface structures than to chitins with flat/smooth film-like surface structures, reflecting both physical and ionic interactions.

**Figure 5:**
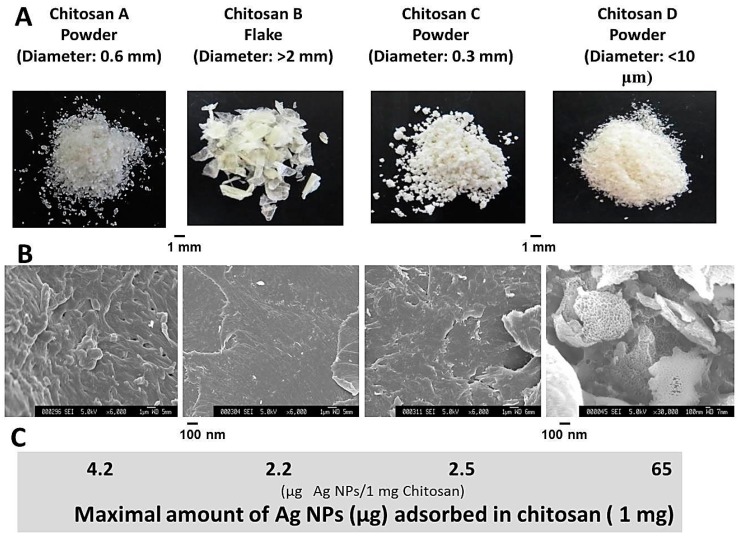
SEM images of chitosan surfaces and binding of Ag NPs. Adsorption of Ag NPs to chitosans with nanoscale porous surface structures was higher than to chitosans with flat/smooth film-like surface structures. (**A**) Appearance of each chitosan powder (flake); (**B**) SEM micrographs of each chitosan; (**C**) Binding of Ag NPs onto each chitosan.

A fine chitosan powder with grain diameters of <10 μm, a nanoscale porous surface structure (chitosan A), and a DDAc of 75% was added as a stabilizer to the Ag NP suspension at pH 7.3 to remove caramel and prevent agglomeration and precipitation of Ag NPs. Under these conditions, a maximum of 65 μg of Ag NPs was adsorbed onto 1 mg of chitosan (Figure 5), and Ag NPs in the ensuing chitosan-Ag NPs composite were substantially stabilized compared with Ag NPs alone, and had similar stability to those bound to chitin with a nanoscale fiber-like surface structure. Moreover, sizes and shapes of Ag NPs in chitosan-Ag NP composites were similar to those of the original Ag NPs (data not shown). However, when coarse chitosan powders with diameters of 0.6 and 0.3 mm (or flakes of >2 mm), a DDAc of 70%, and flat/smooth film-like surface structures (chitosan B, C and D) were used, maximal adsorption of Ag NPs was 4.2, 2.2, and 2.5 μg of Ag NPs per 1 mg of chitosan, respectively. These observations indicate that powder sizes and nanoscale fiber-like or porous surface structures in chitin/chitosan powders may crucially influence adsorption of Ag NPs and antimicrobial activities of chitin/chitosan-Ag NP composites (original manuscript in preparation).

## 3. Antimicrobial Activities of Chitin/Chitosan and Ag NP Composites

Due to the increasing prevalence of antibiotic resistance, antimicrobial activities of Ag NPs have been demonstrated in a number of studies [17,25,30,31]. The dependence of antiviral activities on particle sizes and Ag NP contents was demonstrated [20,24,25] according to 50% tissue culture infectious dose (TCID_50_) ratios of H1N1 Influenza A virus suspensions treated with chitosan-Ag NPs composites. Among small (diameter, 3.48 ± 1.83 nm), medium (6.53 ± 1.78 nm), and large (12.9 ± 2.5 nm) Ag NP nanoparticles, antiviral activities of chitosan-Ag NP composites increased with increasing Ag NP adsorption, and no antiviral activity was observed with chitosan alone. Moreover, at constant Ag NP concentrations, stronger antiviral activity was generally observed with composites containing smaller Ag NPs [20,24].

**Figure 6:**
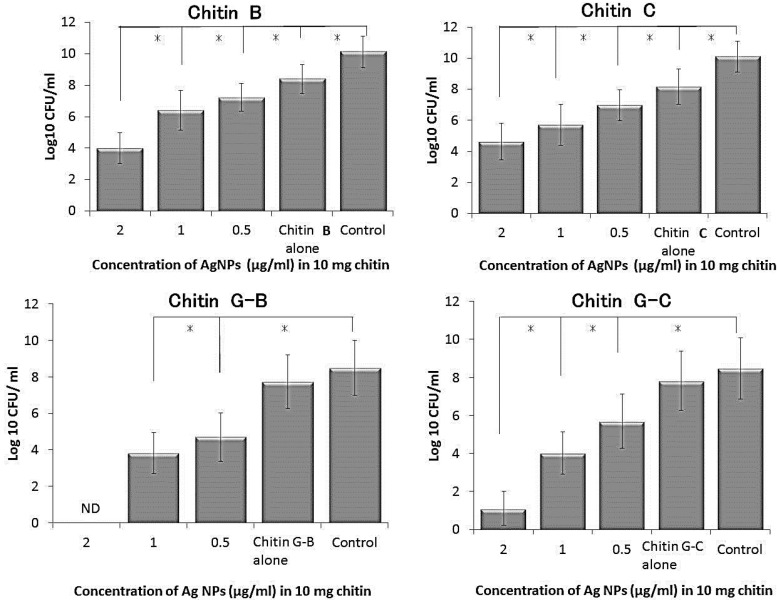
Antibacterial activities of chitin (B, C, G-B, and G-C)-Ag NP composites with various amounts of Ag NPs. Chitins B, C, G-B, and G-C/Ag NP composites were evaluated for their bactericidal (against *E. coli*) activity in LB medium. Chitin B, C, G-B, and G-C (each 10 mg) was interacted with various concentrations of Ag NP-suspensions (0, 0.5, 1 and 2 μg/mL) to prepare each chitin-Ag NP composite. Almost all Ag NPs in the Ag NP suspension were estimated to be adsorbed onto Chitin G-B and G-C, but only partial Ag NPs might be adsorbed onto Chitin B and C. Each composite contained various amounts of Ag NPs (10 mg) of chitin exhibited Ag NP concentration-dependent bactericidal activity in each chitin. Asterisks (*) denote statistically significant differences (*p* < 0.01) as determined using a two-sample *t*-test.

Bactericidal activities of chitin and chitin-Ag NP composites have been evaluated in *E. coli*. In these experiments, individual chitins exhibited only weak bactericidal activity, whereas composites of 10 mg/mL chitins A, G-B, and G-C with various Ag NPs contents showed strong concentration-dependent bactericidal activity. However, composites of chitins B and C had poor activity in these studies, reflecting weak interactions with Ag NPs (Figure 6) [28].

The antifungal activities of chitins and chitin-Ag NP composites were evaluated against *A. niger*. In these studies, fungi were incubated in molten potato dextrose agar (PDA) containing the test materials, and chitin alone (5 mg/mL) exhibited weak antifungal activity. In contrast, 5 mg/mL chitin composites with various Ag NP contents showed strong and concentration-dependent antifungal activity, with half-growth inhibition occurring at Ag NP concentrations of 10, 14, and 15 µg/mL in chitins A, G-B, and G-C, respectively. However, composites containing chitin B and C had low activity, indicating that the antifungal activity of chitin-Ag NP composites increases with adsorbed Ag NPs. The antimicrobial activity of Ag NPs on smaller chitin powder particles with nanoscale fiber-like surface structures was likely enhanced by increased available binding areas for Ag NPs, reflecting greater surface areas and the nanoscale fiber-like surface structures. In addition, chitin-Ag NP composites with higher Ag NP contents interacted more efficiently with microorganisms [24,26,28].

Bactericidal actions of Ag ions are closely related to their protein interactions. In particular, Ag ions bridge protein thiol groups (sulfhydryl, -SH) and disrupt protein functions, ultimately leading to metabolic disaster and death [31,32,33]. Although the bactericidal and antifungal mechanisms of Ag NPs have not been investigated extensively, activities of Ag NPs against gram-negative bacteria such as *E. coli* are reportedly dependent on Ag NP concentrations and the formation of pits in bacterial cell walls [34]. Potentially, this mechanism reflects direct binding of Ag NPs on nanoscale fiber-like surface structures of small chitin powder particles to microbial envelope glycoproteins, and consequent facilitation of membrane-compromising interactions. The potent antifungal activity (against *A. niger*) of chitin-Ag NP composites may also reflect compromised membrane integrity [32,35]. Accordingly, special restrictions of course chitin-Ag NP composites with lower Ag NPs contents may prevent or weaken Ag NPs interactions with microorganisms. In agreement with this principle, composites of fine chitin powders with higher Ag NPs contents interacted more efficiently with microbial cell surfaces [28].

Ag NPs have also been shown to participate in the formation of bactericidal reactive oxygen species (ROS), leading to membrane damage, activation of respiratory lactate dehydrogenase, DNA damage, and ultimately cell death [35].

## 4. Antimicrobial Activities of Chitin Nanofiber Sheets (CNFS) and Ag NP Composites

Biochemical advantages of chitin/chitosan-based materials include anti-infectious activities [1,4], stimulation of angiogenesis/wound repair, and stabilization/activation of growth factors [36,37,38,39]. Moreover, recent interest in chitin/chitosan nanofibers represents their revolutionary potential in nanotechnology [40]. Ifuku *et al.* isolated α-chitin nanofibers from crab shells, which showed uniform widths of 10–20 nm and high aspect ratios [41,42]. Because chitin nanofiber sheets (CNFS, DDAc of about 30%, Beschitin W, Unichika Ltd., Tokyo, Japan) are biodegradable and have large surface–mass ratios, they have industrial, environmental, and pharmaceutical applications as composite materials. Moreover, favorable properties of CNFS-based materials are enhanced with decreasing fiber sizes in the range of 1–100 nm [40]. Positive surface charges of CNFS and chelating activities of chitin acetamindo groups may also play important roles in adsorption of heavy metals and arsenic [43,44].

**Figure 7:**
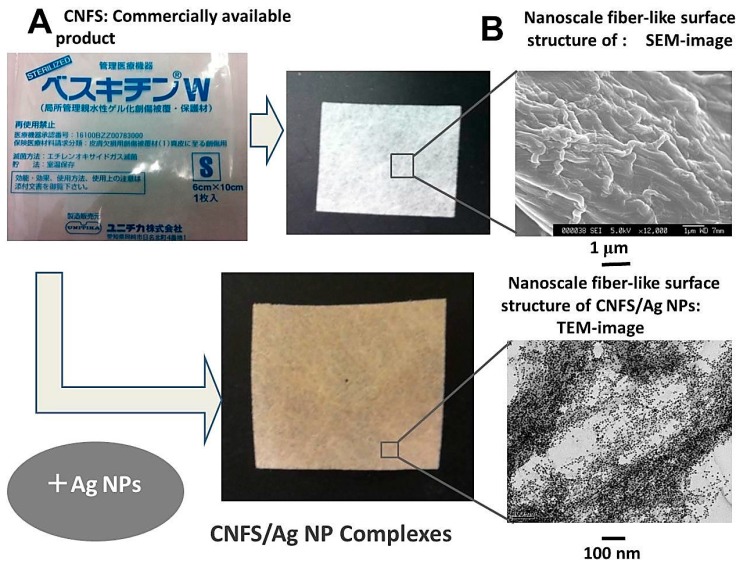
Production (**A**) and appearances (**B**) of chitin nanofiber sheet (CNFS)-Ag NP complexes.

**Figure 8:**
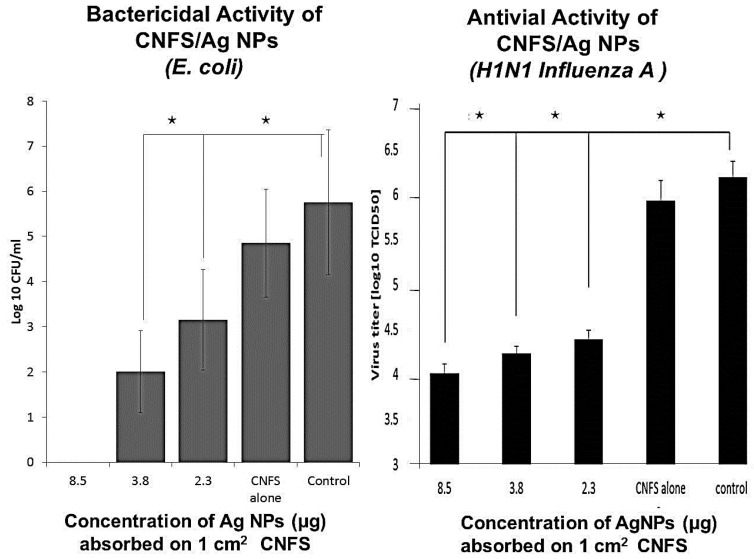
Bactericidal and antiviral activities of various concentrations of Ag NPs on CNFS. Asterisks (*) denote statistically significant differences (*p* < 0.01) as determined using a two-sample *t*-test.

Wound dressings are produced using cotton, chitin, chitosan, alloskin, pigskin, and various other biological materials [45]. However, many of these materials have clinical disadvantages such as low antimicrobial activity, allergenicity, toxic effects, and poor adhesiveness [40,46]. Accordingly, we developed a potential wound dressing, comprising Ag NPs immobilized on CNFS, which may act as a microbial barrier that limits cross contamination (Figure 7). Ag NPs were produced using environment-friendly materials (AgNO_3_-containing glass powder, glucose, and water) and processes that yielded Ag NPs of about 5 nm in diameter as described above. In subsequent experiments, CNFS-Ag NPs showed strong antimicrobial activity against *E. coli* and influenza A virus (Figure 8).

The CNFS used in this study had nanoscale fiber-like surface structures, and correspondingly high surface area availability for adsorption of Ag NPs. Moreover, the anti-infectious [4,47], angiogenesis, wound repair, and growth factor stabilization/activation activities of CNFS have been demonstrated in numerous previous studies [36,37,48,49]. Recent studies show that application of CNFS to skin improves epithelial granular layers and increases granular density, indicating the potential of CNFS in skin-protective formulations [50]. Moreover, α-chitin nanofibrils reportedly have anti-inflammatory and anti-fibrosis effects [51]. In our study, commercially available CNFS was combined with Ag NPs to provide stronger antimicrobial activity, and the potential of this novel composite as a biocompatible wound dressing was examined.

CNFS-Ag NPs showed strong bactericidal activity against *E. coli* and antiviral activity against H1N1 influenza A virus, potentially reflecting interactions between virions and Ag NPs. Accordingly, increasing Ag NPs contents on CNFS may further increase numbers of immobilized virions, yielding increased antiviral activities. Moreover, in the present studies, CNFSs containing 8.5 μg of Ag NPs per 1 cm^2^ of sheet (7.3 ± 0.1 mg) completely eradicated *E. coli*. Several potential mechanisms have been proposed for the bactericidal activities of Ag NPs (Figure 8) [21,22], including decreased density of cytoplasmic components, condensation of bacterial DNA, and detachment of plasma membranes from cell walls. These phenomena suggest that Ag NPs undermine the integrity of the cytoplasm and its membranes, causing malfunction of organelles and leading to cell death. Bacterial DNA may also be damaged by Ag-mediated ROS such as superoxide anions (O_2_^−^) [35], although further biochemical studies are required to characterize these mechanisms. Furthermore, it would be of great interest to measure the same activity for some gram positive strain of bacteria such as *Staphylococcus aureus*, since it was recently demonstrated that a bactericidal activity of AG NPs should be much lower, as bacterial membranes of gram positive are more resistant to the penetration of Ag NPs [52].

The effects of Ag NP size on antiviral activity suggest that various viruses interact selectively with smaller (≤10 nm in diameter) Ag NPs, as previously reported for HIV-1 [53] and hepatitis B viruses [35]. We also reported size-dependence of antiviral effects of free Ag NPs using influenza A virus (Figure 8) [24,25], although spatial restrictions of CNFS may prevent or weaken interactions between virions and Ag NPs. Accordingly, viruses were not completely eradicated after exposure to CNFS-Ag NPs, although adsorption of Ag NPs onto CNFS provided stronger antiviral activity, which may be further increased with increasing Ag NP concentrations on CNFSs.

## 5. Conclusions and Recommendations

Nanoscale surface structures of chitin/chitosan biomaterials adsorb significant quantities of Ag NPs, and have a greater influence on antimicrobial activities of chitin/chitosan-Ag NP composites than do the molecular weights of Ag NPs, DDAc, and structural conformations. In the present studies, various chitin/chitosan powders with nanoscale fiber-like or porous surface structures were used to stabilize Ag NP suspension, to remove the caramel generated during autoclaving, and to prevent aggregation and precipitation of Ag NPs. To this end, Ag NPs were homogenously dispersed and stably adsorbed onto chitin powders or sheets with nanoscale fiber-like surface structures or onto chitosan powders with nanoscale porous surface structures. Subsequent experiments showed that bactericidal, antifungal, and antiviral activities of chitin-Ag NP composites increased with Ag NP adsorption, indicating potential applications of fine chitin powders with nanoscale fiber-like surface structures as novel stabilizers and carriers for Ag NPs. Moreover, chitin/chitosan-Ag NP composites could be used directly as antimicrobial materials. Accordingly, TEM image analyses of CNFS-Ag NPs indicated homogenous dispersion and tight adsorption of Ag NPs, and subsequent antimicrobial assays showed higher antimicrobial activities against *E. coli* and influenza A virus than those of Ag NPs alone.

Finally, the present chitin/chitosan-Ag NPs, and CNFS-Ag NPs show great potential as disinfectant wound dressings, clothes, plastics, and papers, with various applications such as masks, air and water filters, table cloths, and protection coats, *etc.* In fact, Ag NPs could be directly bound to cotton paper and clothes with nanoscale fiber-like surface structures (data not published). In addition, chitin/chitosan with nanoscale fiber-like and porous surface structures may adsorb heavy metals, arsenic, and the other intoxicants.
